# A Matrix-Free 3D Hepatocyte–Macrophage Co-Culture Spheroid Model for Dual Assessment of Lipid Accumulation and NF-κB-Mediated Inflammatory Activation Under Glucolipotoxic Stress

**DOI:** 10.3390/biomedicines14040792

**Published:** 2026-03-31

**Authors:** Federico Ghiselli, Andrea Piva, Ester Grilli

**Affiliations:** Vetagro S.p.A., Via Ignazio Porro 2, 42124 Reggio Emilia, Italy

**Keywords:** MASLD, in vitro disease model, 3D liver spheroids, matrix-free co-culture, hepatocyte–macrophage crosstalk, NF-κB inflammatory signaling, palmitic acid, glucolipotoxicity, macrophage remodeling, steatosis

## Abstract

**Background/Objectives:** Metabolic dysfunction-associated steatotic liver disease (MASLD) involves the interplay of hepatic lipid accumulation and immune-mediated inflammatory signaling, yet human-relevant in vitro systems that capture both processes simultaneously in a scalable format remain limited. The objective of this study was to develop and characterize a matrix-free 3D hepatocyte–macrophage co-culture model enabling simultaneous assessment of lipid accumulation and NF-κB-mediated inflammatory activation under glucolipotoxic stress. **Methods**: A 3D liver co-culture model was established by combining HepG2 hepatocyte-like cells with phorbol 12-myristate 13-acetate (PMA)-differentiated THP-1 macrophage-like cells stably expressing a NF-κB–Luc2 reporter. Spheroids were generated using a hanging-drop method in standard 96-well plates and matured for 8–10 days. Mature spheroids were subjected to acute 24 h glucolipotoxic challenge combining high glucose and palmitic acid and assessed for neutral lipid accumulation, NF-κB reporter activation (luciferase), and macrophage marker expression (qPCR). **Results**: Time-course characterization demonstrated progressive hepatocyte marker remodeling (albumin, alpha-fetoprotein, CYP3A4) and dynamic macrophage phenotype shifts (CD14, CD206, MARCO, TREM2). Acute glucolipotoxic challenge induced dose-dependent increases in neutral lipid accumulation and NF-κB reporter activation, accompanied by coordinated macrophage-associated transcriptional changes consistent with lipid-handling and tissue-remodeling programs. Post-challenge metabolic activity was retained under the selected stress conditions. As a proof-of-concept demonstration, three botanical extracts showed distinct attenuation profiles across the lipid and inflammatory endpoints. **Conclusions**: This 3D hepatocyte–macrophage co-culture model provides orthogonal readouts of steatosis and NF-κB-mediated inflammatory activation under glucolipotoxic stress, offering a reproducible, fit-for-purpose screening tool for investigating early glucolipotoxic hepatic responses and evaluating candidate compounds in a defined in vitro setting.

## 1. Introduction

Metabolic dysfunction-associated steatotic liver disease (MASLD; formerly non-alcoholic fatty liver disease) has overtaken viral hepatitis as the leading cause of chronic liver disease, affecting approximately one-third of adults worldwide, and is expected to increase further in coming decades [1,2]. The 2023 international consensus that adopted the MASLD/MASH terminology emphasized that the disorder is driven by metabolic dysfunction rather than by the mere absence of alcohol exposure [3]. Although simple steatosis is often clinically silent, paired-biopsy meta-analyses indicate that approximately one-third of patients develop inflammatory steatohepatitis (MASH) or clinically significant fibrosis within ten years, with faster progression in individuals with obesity and diabetes [4]. Once inflammation and fibrogenesis are established, liver-related mortality, cardiovascular events and extra-hepatic cancers increase, underscoring the need for interventions that target early, pre-fibrotic disease [5]. Therapeutic options remain limited; in 2024, the U.S. FDA granted accelerated approval to resmetirom (Rezdiffra™) for noncirrhotic MASH with F2–F3 fibrosis, yet no agent is licensed for the much larger population with uncomplicated steatosis [6].

At the molecular level, the progression from simple steatosis to inflammatory steatohepatitis involves multiple converging pathways [7]. Saturated fatty acids, particularly palmitic acid, trigger endoplasmic reticulum stress, mitochondrial dysfunction, and the generation of reactive oxygen species in hepatocytes, which collectively activate the NF-κB signaling cascade and promote the release of pro-inflammatory mediators [8]. Concurrently, hepatic macrophages, both resident Kupffer cells and recruited monocyte-derived populations, serve as central effectors of the innate immune response in the steatotic liver, undergoing phenotypic shifts that influence disease outcome [9]. The interplay between lipid-loaded hepatocytes and activated macrophages is therefore recognized as a critical determinant of MASLD progression, making it an important target for in vitro modeling [9]. Conventional two-dimensional (2D) hepatocyte monolayers are inexpensive and easy to implement, but they rapidly lose polarity, metabolic competence, and immune crosstalk, limiting their utility for modeling early inflammatory activation in metabolically stressed settings [10]. At the other end of the spectrum, liver-on-a-chip and related microphysiological systems can offer high physiological fidelity, but they often depend on scarce primary tissues and specialized platforms that constrain routine use [11]. Recent scaffold-free spheroid models [12], including HepG2/monocyte co-cultures [13], primary-hepatocyte hydrogels [14], hepatocyte/stellate-cell spheroids [15], and quadruple co-cultures [16], have advanced the field by capturing steatosis, inflammation, and fibrosis in human-relevant 3D formats. However, many 3D platforms still rely on proprietary matrices, primary donors, or complex perfusion systems. These requirements increase cost and variability, limiting their use in routine screening settings where multiple conditions or candidate compounds must be compared in parallel.

THP-1 cells can be differentiated reproducibly into a macrophage-like phenotype and, when equipped with an NF-κB luciferase reporter, provide a direct functional readout of inflammatory pathway activation. While primary-cell systems offer higher physiological fidelity, cell-line-based models prioritize standardization and repeatability [17]. Even a cell-line-based 3D approach requires careful optimization of cell ratios, macrophage induction, maturation duration, and assay conditions to achieve consistent spheroid formation and reliable reporter performance. Validation in primary-cell or iPSC-derived systems will be an important next step to further define the biological scope of the model [18].

The objective of the current study was to develop and characterize a simple, matrix-free 3D HepG2/THP-1 (NF-κB-Luc2) co-culture model that matures reproducibly in standard 96-well plates and enables simultaneous quantification of glucolipotoxic responses across two orthogonal endpoints: neutral lipid accumulation (Nile Red/DAPI fluorescence) and inflammatory activation (NF-κB reporter activity). The model is presented as a reproducible, fit-for-purpose screening tool for investigating hepatic lipid–immune interactions under acute glucolipotoxic stress and is not intended to replicate the full complexity of chronic disease progression.

## 2. Materials and Methods

### 2.1. Chemicals and Reagents

Unless otherwise stated, chemicals and cell culture reagents were purchased from Merck Life Science S.r.l. (Milan, Italy). Decaffeinated Green Tea Extract (GTE) was purchased from Plantextrakt GmbH & Co. (Vestenbergsgreuth, Germany). GTE contained 67.50 ± 0.10% (*w*/*w*) total catechins and <0.60 ± 0.10% (*w*/*w*) caffeine. Milk Thistle (MT) was purchased from NUTRIFOODS S.L. (Barcelona, Spain). MT contained 80.22 ± 0.10% (*w*/*w*) silymarin, of which 42.12 ± 0.10% (*w*/*w*) was silybin + isosilybin. Grape Seed Extract (GSE) was purchased from Guilin Layn Natural Ingredients Corp (Guilin, China). GSE contained 89.73 ± 0.10% (*w*/*w*) polyphenols, of which 99.58 ± 0.10% (*w*/*w*) were proanthocyanidins. GTE, MT, and GSE were handled under low-light conditions and prepared as concentrated stock solutions according to supplier recommendations and solubility characteristics. Stock solutions were sterile-filtered (0.22 µm) and stored at −20 °C until use. Working concentrations were obtained by dilution into pre-warmed culture media immediately before treatments, using final concentrations of MT 50 µg/mL, GTE 10 µg/mL, and GSE 10 µg/mL. The final vehicle concentration was kept constant across treatment groups (vehicle-matched controls) and did not exceed 0.10% (*v*/*v*). Stock solutions of β-mercaptoethanol and puromycin were prepared in cell culture-grade water. Nile Red, DAPI (4′,6-diamidino-2-phenylindole), and phorbol 12-myristate 13-acetate (PMA) were dissolved in 100% DMSO at concentrations ensuring a final DMSO ≤ 0.10% (*v*/*v*) in culture medium. Palmitic acid (PA) was prepared in isopropanol; final isopropanol during challenges was 0.10% (*v*/*v*) and vehicle-matched in controls. Fatty-acid-free bovine serum albumin (BSA—low endotoxin) was added at 0.22% (*w*/*v*); PA was pre-complexed for 30 min at 37 °C targeting a 3:1 PA:BSA molar ratio before dilution into medium. Fetal bovine serum (FBS; Merck Life Science, Cat# F7524, Lot# 0001669688) was heat-inactivated (56 °C, 30 min) to inactivate complement. The same lot was used throughout to minimize lot-to-lot variability. All stock solutions were stored at −20 °C until use, except PA stock, which was freshly prepared for each experiment.

### 2.2. Cell Culture and THP-1 Macrophage Induction

This study did not involve human participants, human tissue, or live vertebrate animals. HepG2 cells (HB-8065™) and THP-1 NF-κB-Luc2 (TIB-202-NFkB-LUC2™) were obtained from ATCC^®^ (American Type Culture Collection, Manassas, VA, USA). HepG2 and THP-1 NF-κB-Luc2 were authenticated by STR profiling (ATCC^®^ Lot number 70050519 and 70059144) and were mycoplasma-negative. HepG2 and THP-1 cells were used within a pre-defined passage window (HepG2 between passage 78 and 85 and THP-1 between passage 8 and 18) under a controlled cell-banking strategy to maintain assay stability and inter-run comparability, in line with good practice for cell-based assay standardization [19,20].

HepG2 cells were cultured in Dulbecco’s Modified Eagle’s Medium (DMEM) high glucose (HG—4.5 g/L–25 mM), supplemented with 10% FBS, 1% L-glutamine, 100 U/mL penicillin, 0.10 mg/mL streptomycin, and 1% non-essential amino acids. THP-1 cells were maintained in RPMI 1640 medium supplemented with 10% FBS, 1% L-glutamine, 100 U/mL penicillin, 0.10 mg/mL streptomycin, 1% non-essential amino acids, 10 mM HEPES (4-(2-hydroxyethyl)-1-piperazineethanesulfonic acid), 50 µM β-mercaptoethanol, and 1 µg/mL puromycin. Puromycin was used only for maintenance of the reporter line and was omitted during spheroid maturation and challenge. Both cell types were cultured at 37 °C in a humidified atmosphere containing 5% CO_2_.

Macrophage differentiation was induced following the optimized protocol described by Liu et al. (2023) [21]. Briefly, THP-1 cells were treated with 80 ng/mL PMA at a density of 5 × 10^5^ cells/mL for 24 h, followed by a 24 h rest period in PMA-free medium prior to spheroid seeding. Adherent THP-1 cells induced by PMA were dissociated using an Accutase^®^ solution, washed, and counted to maintain a constant HepG2:THP-1 seeding ratio.

### 2.3. Metabolic Activity Assay, Live/Dead Staining and Morphology

Spheroid metabolic activity was assessed using PrestoBlue™ (Thermo Fisher Scientific, Milan, Italy) according to the manufacturer’s instructions. Fluorescence was measured on a Varioskan™ LUX (Thermo Fisher Scientific, Milan, Italy) using 560/590 nm excitation/emission settings. For time-course analyses, signals were normalized to the starting seeded cell number (3000 or 6000) and reported as normalized RFU. Post-challenge PrestoBlue™ fluorescence was assessed after the 24 h glucolipotoxic exposure and used as an assay-window control to confirm that the selected challenge retained measurable metabolic competence compatible with short-term experimental use.

Brightfield images were taken using a Nikon Eclipse TS100 inverted microscope (Nikon Corporation—Tokyo, Japan) and analyzed with NIS-Elements software 5.0 (Nikon Corporation—Tokyo, Japan). Live/Dead staining was performed using the LIVE/DEAD™ Cell Imaging Kit (488/570-Thermo Fisher Scientific, Milan, Italy) according to the manufacturer’s guidelines. Spheroids were transferred to microscope slides and mounted with a fluoroshield containing 4′,6-diamidine-2′-phenylindole dihydrochloride (DAPI). Images were acquired using a Nikon Eclipse Ci upright fluorescence microscope at 20× magnification and analyzed with NIS-Elements software.

### 2.4. Immunofluorescence Staining

Immunofluorescence (IF) staining for albumin (ALB) and CD14 was conducted following the methodology described by Ghiselli et al., 2021 [22]. In summary, after 10 days spheroids were fixed with 4% paraformaldehyde in DPBS for 20 min and then permeabilized using 0.5% Triton X-100 (VWR, Radnor, PA, USA) for 15 min. To block nonspecific binding, spheroids were incubated with 10% goat serum for 1 h. Primary monoclonal antibodies (listed in Table 1) were diluted in DPBS containing 2% BSA and 0.05% saponins (Alfa Aesar, Haverhill, MA, USA) and applied for 3 h at room temperature in a humidified environment. Detection was performed using fluorescein isothiocyanate- or tetramethylrhodamine-conjugated secondary antibodies for 1 h, with dilutions specified in Table 1. After two washes with DPBS supplemented with 0.2% BSA and 0.05% saponins, spheroids were visualized. Spheroids were transferred to microscope slides and mounted with a fluoroshield containing 4′,6-diamidine-2′-phenylindole dihydrochloride (DAPI). Images were acquired using a Nikon Eclipse Ci upright fluorescence microscope at 20× magnification and analyzed with NIS-Elements software.

### 2.5. qPCR

Total RNA was isolated from stimulated spheroids using the NucleoSpin RNA kit (Macherey-Nagel Inc., Bethlehem, PA, USA), in accordance with the manufacturer’s guidelines. RNA quantity and purity were evaluated spectrophotometrically by measuring absorbance at 260 and 280 nm with a Varioskan™ LUX instrument (Thermo Fisher Scientific, Milan, Italy). Only samples exhibiting a 260/280 absorbance ratio of 1.8 or higher were included in downstream analyses.

Subsequently, reverse transcription of RNA into cDNA was carried out using the iScript cDNA synthesis kit (Bio-Rad Laboratories, Hercules, CA, USA), following the supplier’s instructions.

Quantitative PCR (qPCR) was run on a CFX Connect using iTaq™ Universal SYBR^®^ Green Supermix (Bio-Rad Laboratories, Hercules, CA, USA) with the manufacturer’s cycling program: 95 °C polymerase activation/initial denaturation for 30 s, followed by 40 cycles of 95 °C for 5 s and 60 °C for 30 s with plate read. A post-amplification melt curve was acquired from 55–95 °C in 0.5 °C increments (5 s/step) to verify single-product amplification. Primer efficiencies were within 90–100%; therefore the 2^−ΔΔCt^ method was applied [23]. The working cDNA input for experimental samples was set at 10 ng per 10 µL reaction.

For the spheroid characterization experiments, fold changes were calculated using 2D monolayers as calibrators (2D HepG2 for hepatocyte genes; 2D PMA-induced-THP-1 for macrophage genes). For MASLD induction experiments, fold changes were calculated relative to the low-glucose (LG) group. Unless otherwise stated, 2D calibrators were cultured in parallel and harvested at the same time points as spheroids. Melting curves showed single peaks and no primer-dimers. RNA was extracted from intact spheroids by design to capture an integrated co-culture response suitable for systematic comparisons across conditions. Amplicon sizes matched predictions, and reference genes (RPL13, TBP) were stable across conditions. Information on primer sequences, expected amplicon sizes, and corresponding GenBank accession numbers is available in Table 2. Primers were designed using the Primer-BLAST tool (https://www.ncbi.nlm.nih.gov/tools/primer-blast/ accessed 2 March 2024) and synthesized by Merck Life Science S.r.l.

### 2.6. Glucolipotoxic Challenge and Bioactive Compound Testing

Spheroids were maintained for 8 days in LG medium (5.5 mM glucose) prior to challenge. On Day 8, spheroids were gently washed in pre-warmed DPBS and assigned to experimental conditions for a 24 h exposure. Basal challenge conditions included: LG control, HG (25 mM glucose), LG + PA, and HG + PA. For palmitic acid dose–response experiments, PA was tested at 0.04, 0.06, 0.08, and 0.10 mM in both LG and HG media. All treatment media contained 0.22% (*w*/*v*) fatty-acid-free BSA. PA was pre-complexed with BSA at 37 °C for 30 min targeting a 3:1 PA:BSA molar ratio prior to dilution into media. Isopropanol concentration was kept constant across conditions (final 0.10% *v*/*v*). For the botanical proof-of-concept assay, spheroids were challenged using a glucolipotoxic positive-control condition (HG + 0.10 mM PA) and treated in parallel with MT (50 µg/mL), GTE (10 µg/mL), or GSE (10 µg/mL) for 24 h. The corresponding negative control was maintained in LG medium. Vehicles were kept constant across groups. Extract-only assay blanks were included to monitor potential optical interference from complex extracts. Metabolic competence under the selected positive-control condition was verified during glucolipotoxic challenge optimization.

### 2.7. Lipid Accumulation Assay

Spheroids were exposed to the respective treatment conditions for 24 h at 37 °C to model glucolipotoxic stress. At the end of the challenge, spheroids were gently washed twice with pre-warmed DPBS to remove residual media. Nile Red/DAPI was selected as a high-throughput, widely adopted neutral-lipid screening readout for multiwell liver models, enabling consistent comparative ranking within plates [24,25]. Lipid staining was performed by incubating spheroids for 15 min at 37 °C with a Nile Red-staining solution (2.5 µg/mL). Following staining, spheroids were washed twice with pre-warmed DPBS and then fixed in 4% paraformaldehyde for 20 min at room temperature. After fixation, two additional DPBS washes were performed, and nuclei were stained with a DAPI-containing solution (1 µg/mL) for 15 min at room temperature. Excess DAPI was removed, and spheroids were maintained in DPBS, inside a 96-well plate, until fluorescence measurements. Fluorescence intensity was recorded using a Varioskan™ LUX multimode microplate reader. Nile Red signal was detected using excitation/emission wavelengths of 510/583 nm, while DAPI fluorescence was measured at 358/461 nm. Lipid accumulation was expressed as the Nile Red/DAPI ratio and normalized to the reference condition specified in the figure legends. DAPI fluorescence was used as a proxy for nuclear content to improve intra-plate comparability.

### 2.8. NF-κB Activation Assay

Following the 24 h treatment challenge, spheroids from each condition were collected and transferred into 1.5 mL microcentrifuge tubes. Samples were centrifuged at 800× *g* for 5 min, and the supernatant was discarded. Spheroids were then washed once with DPBS and centrifuged again at 800× *g* for 5 min, and the wash solution was carefully removed. Washed spheroid pellets were then lysed to obtain whole-spheroid lysates. Luciferase activity was quantified using the Pierce™ Firefly Luciferase Glow Assay Kit (Thermo Fisher Scientific, Milan, Italy) according to the manufacturer’s instructions, with minor modifications. After reagent addition, samples were incubated for 15 min at 37 °C under constant agitation, and luminescence was measured using a Varioskan™ LUX multimode microplate reader (Thermo Fisher Scientific, Milan, Italy). Luminescence values were background-subtracted using reagent-only blanks.

### 2.9. Statistics and Reproducibility

The figure legends detail sample sizes and statistical methods. All conditions were processed in parallel under identical handling. Data shown are from one representative experiment (*n* = 8 wells/condition) unless otherwise stated. For fluorescence and luciferase assays, error bars represent the SD of within-experiment well replicates (*n* = 8). For qPCR, each condition was quantified from four independent biological replicates, each generated by pooling two spheroids per replicate (2 spheroids/replicate; *n* = 4 replicates/condition). For qPCR figures, error bars represent the SD of biological replicates from the representative experiment shown. Experiments were repeated independently three times with comparable results. Data are shown as mean ± SD. Normality was assessed by Shapiro–Wilk (*p* > 0.05) and homogeneity of variance by Levene’s test. Time-course metabolic activity data were analyzed by two-way ANOVA with Šídák’s multiple comparisons. Dose–response datasets were analyzed by two-way ANOVA (factors: glucose condition and PA concentration) with Šídák’s multiple comparisons. Single-factor comparisons (e.g., botanical extract testing) were analyzed by one-way ANOVA with Tukey’s post hoc test. A *p* value < 0.05 was considered significant. Exact *p*-values are reported in the text where available; for multi-group figure comparisons, significance is conveyed by letter groupings (*p* < 0.05). Analyses were conducted in GraphPad Prism 10.5.

## 3. Results

### 3.1. Spheroid Formation and Metabolic Activity over Time

**Figure 1:**
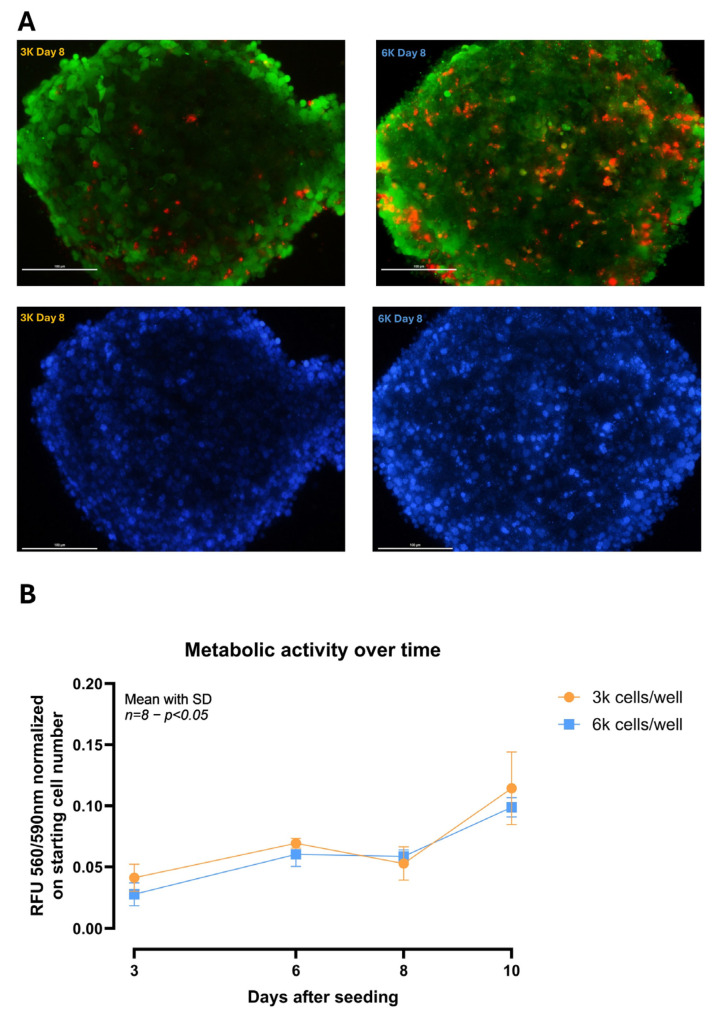
Morphology and metabolic activity of HepG2/THP-1 spheroids at different seeding densities. (**A**) Representative Live/Dead staining at Day 8 of co-culture spheroids seeded at 3000 (3k) or 6000 (6k) total cells/well. Viable cells are calcein-AM-positive (green) and dead cells are EthD-1-positive (red); corresponding nuclear counterstaining (DAPI, blue) is shown in the lower panels. Scale bars: 100 µm. (**B**) Metabolic activity during maturation (Days 3, 6, 8, and 10) measured by PrestoBlue™ fluorescence (Ex/Em 560/590 nm), normalized to the starting seeded cell number, and reported as normalized RFU. The graph shows one representative independent experiment; data are presented as mean ± SD of 8 technical replicate wells per condition. The experiment was repeated independently three times with comparable results. Two-way ANOVA (factors: time and seeding density) with Šídák’s multiple comparisons. Abbreviations: RFU, relative fluorescence units; EthD-1, ethidium homodimer-1; DAPI, 4′,6-diamidino-2-phenylindole.

HepG2/THP-1 co-cultures rapidly self-assembled into compact 3D spheroids using the flipped-plate hanging-drop method. Live/Dead staining at Day 8 showed predominantly viable cells (calcein-AM–positive, green) with sparse dead cells (EthD-1–positive, red) at both seeding densities (3k and 6k), with no evident necrotic core (Figure 1A). Metabolic activity assessed by PrestoBlue™ increased over time in both conditions, with no significant differences between seeding densities across the time course (Figure 1B). Morphologically, 3k spheroids transitioned from looser aggregates at Day 3 to compact near-spherical structures by Days 6–8, maintaining integrity through Day 10, whereas 6k spheroids compacted earlier and appeared larger with smoother edges (Supplementary Figure S1). Based on handling and spheroid size compatible with diffusion-limited viability and plate-based readouts, 3k spheroids were selected as the assay-optimized operating point for subsequent experiments.

### 3.2. Hepatic and Macrophage Marker Expression During Phenotypic Maturation

**Figure 2:**
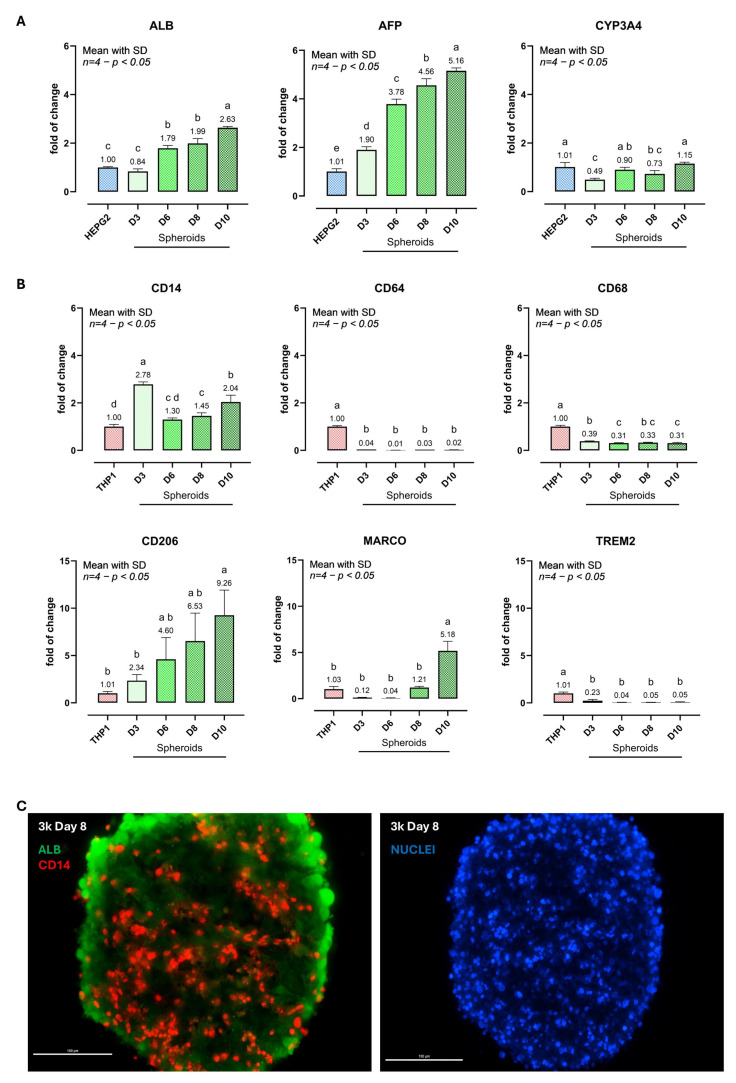
Hepatocyte and macrophage marker expression during spheroid phenotypic remodeling. HepG2/THP-1 spheroids were harvested at Days 3, 6, 8, and 10 for bulk-spheroid RNA extraction followed by qPCR. (**A**) Hepatocyte-associated transcripts (ALB, AFP, CYP3A4) expressed as fold change versus 2D HepG2 monolayers (calibrator = 1.00). (**B**) Macrophage-associated transcripts (CD14, CD64, CD68, CD206, MARCO, TREM2) expressed as fold change versus 2D PMA-differentiated THP-1 cells (calibrator = 1.00). (**C**) Representative immunofluorescence at Day 8 showing albumin (ALB, green) and CD14 (red); nuclei counterstained with DAPI (blue). Scale bars: 100 µm. The graph shows one representative independent experiment; data are presented as mean ± SD of *n* = 4 biological replicates per condition, each biological replicate generated by pooling 2 spheroids. The experiment was repeated independently three times with comparable results. One-way ANOVA with Tukey’s multiple comparisons; groups not sharing letters differ significantly (*p* < 0.05). Abbreviations: ALB, albumin; AFP, alpha-fetoprotein; CYP3A4, cytochrome P450 family 3 subfamily A member 4; CD, cluster of differentiation; MARCO, macrophage receptor with collagenous structure; TREM2, triggering receptor expressed on myeloid cells 2; DAPI, 4′,6-diamidino-2-phenylindole.

To characterize time-dependent maturation, hepatocyte- and macrophage-associated markers were quantified by bulk-spheroid RNA extraction followed by qPCR across Days 3–10 and expressed as fold change versus 2D calibrators (2D HepG2 for hepatocyte markers; 2D PMA-differentiated THP-1 for macrophage markers) (Figure 2). Hepatic transcripts showed progressive remodeling (Figure 2A). ALB increased from 0.84-fold at Day 3 to 1.79-fold (Day 6), 1.99-fold (Day 8), and 2.63-fold (Day 10) versus 2D HepG2 (one-way ANOVA *p* < 0.0001; significant differences as indicated by letter groupings). Alpha-fetoprotein (AFP) increased from 1.90-fold (Day 3) to 3.78-fold (Day 6), 4.56-fold (Day 8), and 5.16-fold (Day 10) (*p* < 0.0001). CYP3A4 decreased early (0.49-fold at Day 3), partially recovered at Day 6–8 (0.90- and 0.73-fold), and returned near baseline by Day 10 (1.15-fold) (*p* < 0.0001).

Macrophage markers confirmed THP-1 incorporation and revealed dynamic shifts (Figure 2B). CD14 peaked at Day 3 (2.78-fold), decreased at Days 6–8 (1.30–1.45-fold), and increased again by Day 10 (2.04-fold) versus 2D PMA-THP-1 (*p* < 0.0001). In contrast, CD64 remained strongly reduced throughout (0.01–0.04-fold), and CD68 was also consistently lower (0.31–0.39-fold) relative to the 2D calibrator (both *p* < 0.0001). Markers associated with macrophage remodeling increased over time: CD206 rose from 2.34-fold (Day 3) to 4.60-fold (Day 6), 6.53-fold (Day 8), and 9.26-fold (Day 10), while MARCO remained low at Days 3–6 (0.12–0.04-fold), approached baseline at Day 8 (1.21-fold), and increased to 5.18-fold at Day 10 (*p* < 0.0001). TREM2 stayed reduced across the time course (0.23–0.05-fold; *p* < 0.0001). Immunofluorescence at Day 8 supported the co-culture architecture (Figure 2C): ALB-positive cells were distributed throughout the spheroid, while CD14-positive cells appeared more frequently toward the periphery, with occasional cells observed within the core. Taken together, the time-dependent marker profiles indicate that the 3D co-culture environment promotes progressive hepatocyte functional maturation alongside dynamic macrophage phenotypic adaptation, establishing a molecularly characterized baseline for subsequent glucolipotoxic challenge experiments.

### 3.3. Glucolipotoxic Challenge: Palmitic Acid Dose–Response Under Low vs. High Glucose

**Figure 3:**
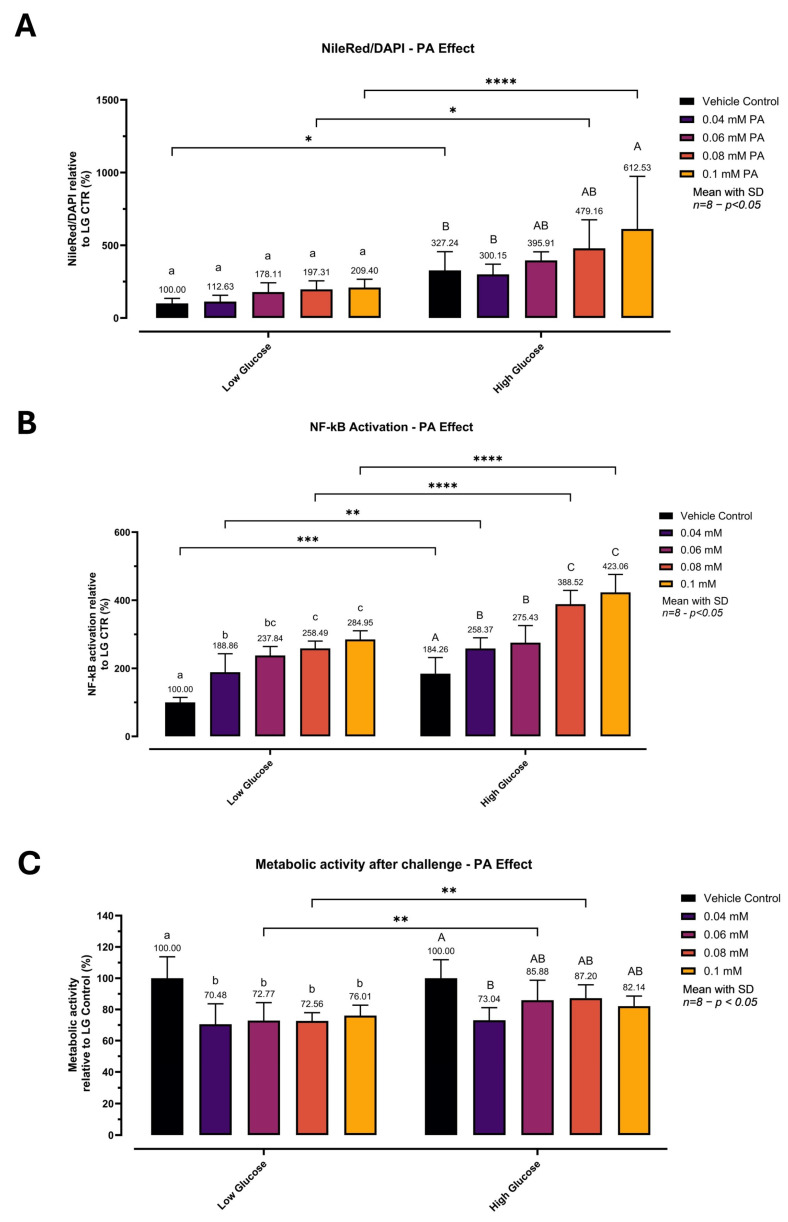
Optimization of acute glucolipotoxic challenge: palmitic acid dose–response under low vs. high glucose. Spheroids were matured for 8 days in low glucose (LG, 5.5 mM) and then exposed for 24 h to palmitic acid (PA; 0.04–0.10 mM) under LG or high-glucose (HG, 25 mM) conditions. PA was BSA-complexed (0.22% *w*/*v* fatty-acid-free BSA; target 3:1 PA:BSA molar ratio), and the vehicle was matched across conditions. (**A**) Neutral lipid accumulation measured as Nile Red/DAPI and expressed as % relative to the LG vehicle control within the same plate (LG vehicle = 100%). (**B**) NF-κB activation measured as luciferase activity and expressed as % relative to the LG vehicle control within the same plate (LG vehicle = 100%); luminescence was background-subtracted using reagent-only blanks. (**C**) Post-challenge metabolic activity assessed by PrestoBlue™ fluorescence and expressed as % relative to the LG vehicle control within the same experiment (LG vehicle = 100%). The graph shows one representative independent experiment; data are presented as mean ± SD of 8 technical replicate wells per condition. The experiment was repeated independently three times with comparable results. Two-way ANOVA (factors: glucose condition and PA concentration) followed by Šídák’s multiple comparisons. Lowercase letter groupings indicate significant differences among PA concentrations within LG, and uppercase letter groupings indicate significant differences within HG (Šídák-adjusted, *p* < 0.05). Brackets/asterisks indicate LG vs. HG comparisons at the same PA concentration (* *p* < 0.05, ** *p* < 0.01, *** *p* < 0.001, **** *p* < 0.0001). Abbreviations: LG, low glucose; HG, high glucose; PA, palmitic acid; BSA, bovine serum albumin; DAPI, 4′,6-diamidino-2-phenylindole.

A glucolipotoxic challenge was established by testing a PA dose–response (0.04–0.10 mM) under LG and HG conditions (Figure 3). In Figure 3A, neutral lipid accumulation (Nile Red/DAPI) was expressed relative to the LG vehicle control. Overall, lipid content was strongly higher in HG than LG (main glucose effect: *p* < 0.0001). Within LG, PA increased Nile Red/DAPI up to 209.40% at 0.10 mM, but no pairwise differences among PA concentrations reached significance. Within HG, baseline lipid content was elevated (327.24%) and increased further with PA, reaching 612.53% at 0.10 mM. In Figure 3B, NF-κB reporter activity increased with both glucose and PA. Two-way ANOVA showed a significant glucose × PA interaction (*p* = 0.0046) and significant main effects of glucose and PA (both *p* < 0.0001). Within LG, NF-κB increased progressively from vehicle to PA doses (letters). HG alone increased NF-κB versus LG, and PA further augmented activation under HG, peaking at 423.06% at 0.10 mM. As shown in Figure 3C, post-challenge metabolic activity (PrestoBlue™) decreased in response to PA (main PA effect: *p* < 0.0001) and differed by glucose condition (*p* = 0.0002), without a significant interaction (*p* = 0.1648). Within LG, all PA doses reduced metabolic activity versus vehicle. Under HG, metabolic activity decreased at 0.04 mM and did not decrease further at higher PA concentrations. As PrestoBlue™ reflects overall cellular reducing capacity, the post-challenge signal was used as an assay-window control to verify that the selected HG + 0.10 mM PA condition retained sufficient metabolic activity for experimental purposes, rather than as a measure of pathway-specific mitochondrial effects. Dose-dependent separation was most evident for NF-κB activity under high-glucose conditions, whereas lipid accumulation in LG showed higher variability despite an increasing trend. The coordinated, dose-responsive increases in both lipid accumulation and NF-κB-mediated inflammatory activation suggest that the model captures key features of acute glucolipotoxic stress in vitro and provides an adequate dynamic range for compound evaluation. Reported statistical values correspond to the representative experiment shown; trends were consistent across three independent runs. Based on these data, HG + 0.10 mM PA was selected as the reference stress condition for subsequent experiments, as it produced the strongest combined response while maintaining measurable metabolic activity.

### 3.4. Cytokine and Metabolic Gene Expression Under Glucolipotoxic Conditions

**Figure 4:**
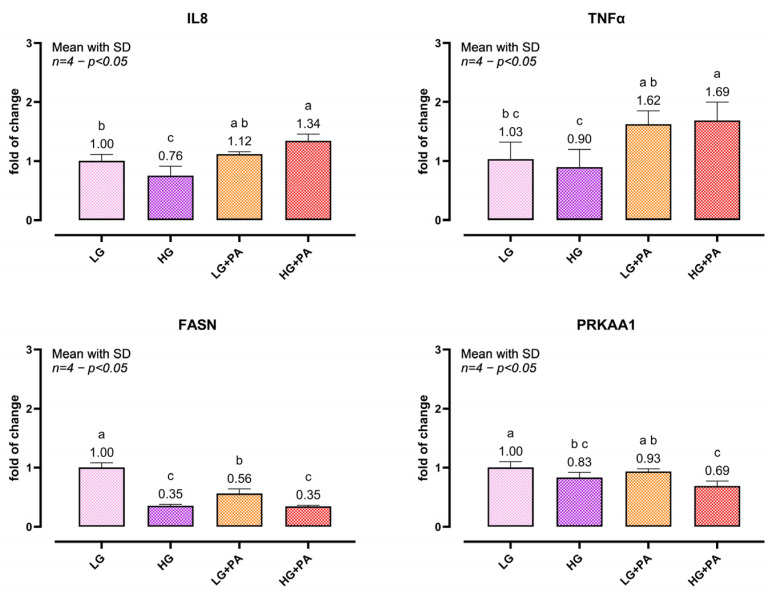
Cytokine and metabolic gene expression under glucolipotoxic challenge. Spheroids were exposed for 24 h to low glucose (LG), high glucose (HG), LG + palmitic acid (PA, 0.10 mM), or HG + PA (0.10 mM) and processed for bulk-spheroid RNA extraction followed by qPCR. Relative mRNA expression of IL8, TNFα, FASN, and PRKAA1 is shown as fold change relative to the LG group, using RPL13 and TBP as reference genes. The graph shows one representative independent experiment; data are presented as mean ± SD of *n* = 4 biological replicates per condition, each biological replicate generated by pooling 2 spheroids. The experiment was independently repeated three times with comparable results. One-way ANOVA within each gene followed by Tukey’s multiple comparisons. Bars not sharing letters differ significantly (*p* < 0.05). Abbreviations: IL8, interleukin-8; TNFα, tumor necrosis factor alpha; FASN, fatty acid synthase; PRKAA1, protein kinase AMP-activated catalytic subunit alpha 1; LG, low glucose; HG, high glucose; PA, palmitic acid; RPL13, ribosomal protein L13; TBP, TATA-box binding protein.

Cytokine- and metabolism-related transcripts after 24 h exposure to LG, HG, LG + PA (0.10 mM), or HG + PA (0.10 mM) are shown in Figure 4. IL8 was reduced under HG compared with LG (*p* = 0.0418), while HG + PA increased IL8 relative to HG (*p* = 0.0035). LG + PA showed an intermediate response. TNFα was not altered by HG alone compared with LG, whereas PA increased TNFα, with the highest levels in HG + PA (*p* = 0.0092 LG vs. LG + PA; *p* = 0.0161 LG vs. HG + PA). FASN was markedly downregulated under HG (*p* < 0.0001) and HG + PA (*p* < 0.0001), with an intermediate decrease in LG + PA (*p* = 0.0016). PRKAA1 showed a modest reduction under HG (*p* = 0.0498) and the strongest decrease under HG + PA (*p* = 0.0007).

### 3.5. Macrophage Marker Expression Under Glucolipotoxic Conditions

**Figure 5:**
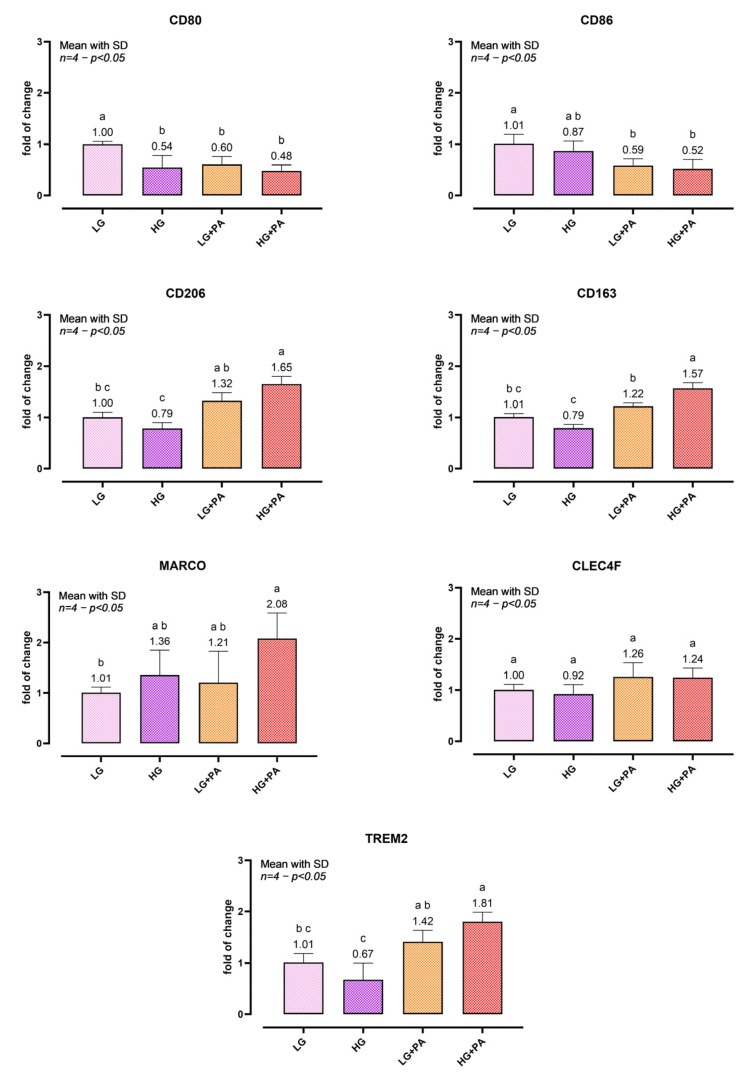
Macrophage marker expression under glucolipotoxic challenge. Spheroids were exposed for 24 h to low glucose (LG), high glucose (HG), LG + palmitic acid (PA, 0.10 mM), or HG + PA (0.10 mM) and processed for bulk-spheroid RNA extraction followed by qPCR. Relative mRNA expression of CD80, CD86, CD163, CD206, MARCO, CLEC4F, and TREM2 are shown as fold change relative to the LG group, using RPL13 and TBP as reference genes. The graph shows one representative independent experiment; data are presented as mean ± SD of *n* = 4 biological replicates per condition, each biological replicate generated by pooling 2 spheroids. The experiment was independently repeated three times with comparable results. One-way ANOVA within each gene followed by Tukey’s multiple comparisons. Bars not sharing letters differ significantly (*p* < 0.05). Abbreviations: CD, cluster of differentiation; CLEC4F, C-type lectin domain family 4 member F; MARCO, macrophage receptor with collagenous structure; TREM2, triggering receptor expressed on myeloid cells 2; LG, low glucose; HG, high glucose; PA, palmitic acid; RPL13, ribosomal protein L13; TBP, TATA-box binding protein.

Macrophage-associated transcripts under glucolipotoxic challenge are shown in Figure 5. CD80 was reduced in all challenged conditions relative to LG, with no differences among HG (*p* = 0.0061 vs. LG), LG + PA (*p* = 0.0161 vs. LG) and HG + PA (*p* = 0.0022 vs. LG). CD86 showed a PA-dependent decrease, with LG + PA (*p* = 0.0206 vs. LG) and HG + PA lower than LG (*p* = 0.0080 vs. LG), while HG alone was intermediate. Markers associated with remodeling/lipid handling showed a graded response: CD206, CD163, MARCO, and TREM2 reached their highest levels under HG + PA, whereas HG alone tended to reduce CD206/CD163/TREM2 relative to LG. CLEC4F was unchanged across conditions. These coordinated transcriptional changes suggest that glucolipotoxic stress triggers a macrophage phenotypic program characterized by downregulation of classical activation markers (CD80, CD86) and concurrent upregulation of receptors associated with lipid scavenging and tissue remodeling (CD206, MARCO, CD163, TREM2), consistent with in vivo observations in MASLD liver biopsies [26].

### 3.6. Proof-of-Concept: Model Responsiveness to Botanical Bioactive Compounds

**Figure 6:**
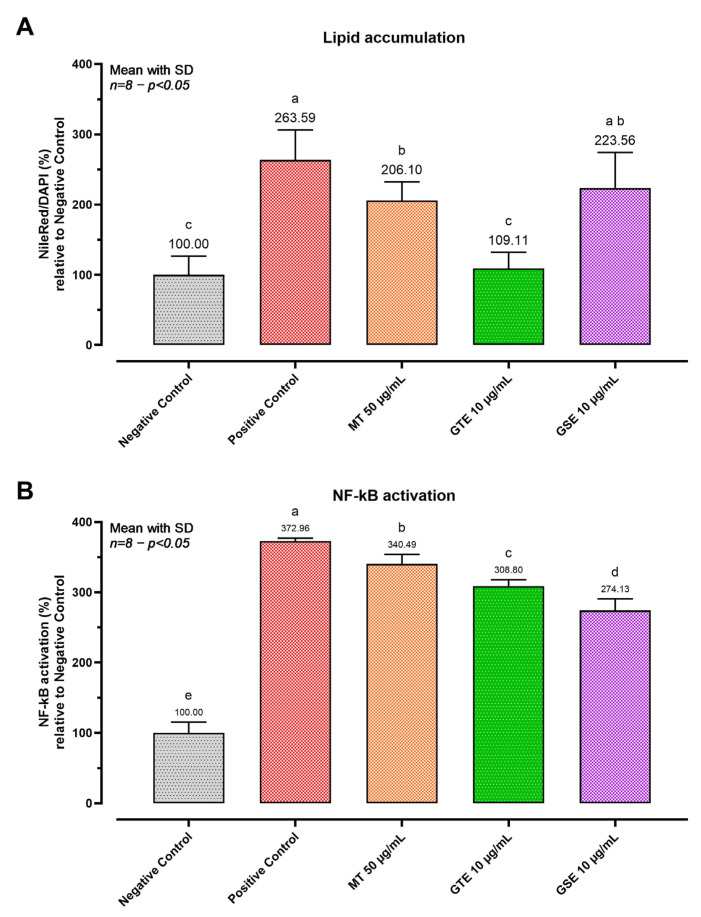
Proof-of-concept model responsiveness to botanical bioactive compounds under glucolipotoxic stress. Spheroids were challenged for 24 h with the glucolipotoxic positive-control condition (HG + 0.10 mM PA) and treated in parallel with Milk Thistle (MT, 50 µg/mL), Green Tea Extract (GTE, 10 µg/mL), or Grape Seed Extract (GSE, 10 µg/mL). The negative control was LG (set to 100%). The vehicle was matched across groups. (**A**) Neutral lipid accumulation quantified as Nile Red/DAPI and expressed as % of the negative control within the same plate (LG = 100%). (**B**) NF-κB activation quantified as luciferase activity and expressed as % of the negative control within the same plate (LG = 100%); luminescence was background-subtracted using reagent-only blanks. The graph shows one representative independent experiment; data are presented as mean ± SD of 8 technical replicate wells per condition. The experiment was repeated independently three times with comparable results. One-way ANOVA within each endpoint followed by Tukey’s multiple comparisons. Bars not sharing letters differ significantly (*p* < 0.05). Abbreviations: LG, low glucose; HG, high glucose; PA, palmitic acid; MT, milk thistle; GTE, green tea extract; GSE, grape seed extract; DAPI, 4′,6-diamidino-2-phenylindole.

To demonstrate model responsiveness to bioactive compounds with reported hepatoprotective properties, a proof-of-concept experiment was performed using the selected glucolipotoxic stress condition (HG + 0.10 mM PA) (Figure 6). Relative to the negative control (LG = 100%), the stress condition markedly increased lipid accumulation (263.59%) and NF-κB activity (372.96%). Under challenge, GTE reduced lipid accumulation to near-baseline levels (109.11%; not different from LG) and partially decreased NF-κB activation (308.80%; *p* < 0.0001). MT produced an intermediate reduction in lipid accumulation (206.10%; *p* < 0.0001) and a modest decrease in NF-κB (340.49%; *p* = 0.0002). GSE did not significantly reduce lipid accumulation relative to the positive control (223.56%) but produced the strongest attenuation of NF-κB activation (274.13%; *p* < 0.0001). To complement the normalized presentation, representative raw signal values were also reported. In the NF-κB luciferase assay, raw luminescence increased from 108 ± 29 RLU in the LG condition to 454 ± 47 RLU under the HG + PA challenge condition, corresponding to an approximately 4.2-fold signal window. In the Nile Red assay, raw fluorescence increased from 2.98 ± 1.38 RFU in LG control spheroids to 8.56 ± 4.65 RFU in HG + PA-treated spheroids, while the Nile Red/DAPI ratio increased from 0.54 ± 0.26 to 1.07 ± 0.71. The higher variability of the Nile Red signal is consistent with the biological heterogeneity of lipid accumulation across individual spheroids and supports the use of normalized ratios for inter-condition comparisons.

These divergent response profiles demonstrate that the model can resolve distinct activity patterns across the lipid and inflammatory endpoints, supporting its utility for comparative evaluation of bioactive compounds targeting different aspects of glucolipotoxic hepatic injury.

## 4. Discussion

This work describes the development and characterization of a matrix-free, immune-responsive 3D HepG2/THP-1 co-culture model providing two orthogonal readouts, neutral lipid accumulation and NF-κB-mediated inflammatory activation, under standardized glucolipotoxic challenge conditions. The model was designed to capture the hepatocyte–macrophage interplay that is increasingly recognized as a central driver of MASLD progression, within a reproducible, cell-line-based format compatible with standard multiwell plates.

### 4.1. Molecular Characterization of the Co-Culture Microenvironment

During maturation, the 3D co-culture environment promoted progressive remodeling of both hepatocyte and macrophage transcriptional programs. Hepatocyte-associated markers showed patterns consistent with 3D reorganization: ALB increased progressively, compatible with enhanced hepatic differentiation in scaffold-free spheroids [27], while the transient CYP3A4 decrease followed by partial recovery aligns with published observations of early remodeling in HepG2 aggregates [28]. AFP upregulation is expected in HepG2-based systems [29] and reflects the hepatocellular carcinoma origin of these cells, representing a known limitation of this cell line. Nevertheless, the co-culture architecture appears to provide relevant contextual cues even within a cell-line-based system. Macrophage marker dynamics were particularly informative. The progressive increase in CD206 and MARCO alongside reduced CD64 and CD68 suggests that the 3D co-culture microenvironment drives phenotypic adaptation of THP-1-derived macrophages toward tissue-remodeling and lipid-handling programs, distinct from the classical phenotype in 2D culture. This is consistent with reports showing that the hepatic microenvironment shapes macrophage identity in vivo [30].

### 4.2. Glucolipotoxic Stress Recapitulates Key Features of Early MASLD

Acute combined exposure to high glucose and palmitic acid induced coordinated, dose-dependent increases in lipid accumulation and NF-κB activation, reproducing two key features of early glucolipotoxic stress in vitro. The observation that PA-induced NF-κB activation was potentiated under high-glucose conditions is consistent with the known synergistic effects of glucolipotoxicity on hepatic inflammatory signaling, whereby hyperglycemia amplifies saturated fatty acid-induced oxidative stress and NF-κB pathway engagement [31]. The transcriptional profile under challenge was inconsistent with classical M1 activation and instead pointed to coordinated remodeling toward lipid-handling and tissue repair programs. This profile shows similarities to the lipid-associated macrophage (LAM) signature described in murine and human MASLD tissues [32,33]. This is consistent with the model capturing both hepatocyte-driven lipotoxic responses and macrophage-associated transcriptional changes under glucolipotoxic stress. Accordingly, the present data should be interpreted primarily as functional assay-development evidence rather than as a comprehensive molecular dissection of inflammatory signaling.

### 4.3. Proof-of-Concept Responsiveness to Bioactive Compounds

The three botanical extracts produced distinct activity profiles across the two endpoints, showing the model’s ability to discriminate anti-steatotic from anti-inflammatory activity within the same experiment. GTE showed the strongest lipid-lowering effect with modest NF-κB attenuation, GSE showed preferential anti-inflammatory activity, and MT exhibited intermediate effects on both endpoints. These profiles are consistent with known pharmacological properties: catechins in green tea are associated with inhibition of lipogenesis and enhanced fatty acid oxidation [32], while proanthocyanidins in grape seed extract are reported to modulate NF-κB signaling directly [34]. Although these data serve as a demonstration of model sensitivity rather than a comprehensive pharmacological evaluation, they illustrate the added value of dual-endpoint profiling in distinguishing mechanistically distinct activities that would be missed by single-readout assays.

### 4.4. Limitations and Future Directions

DAPI fluorescence was used as a nuclear proxy to improve intra-plate comparability of Nile Red signal. While this is a widely used approach in multiwell liver models, treatment-induced changes in nuclear morphology or cell numbers not observed under the conditions used here could theoretically affect the ratio. Future studies may further strengthen normalization robustness by integrating complementary approaches, such as total protein normalization or alternative nuclear markers. Results should therefore be interpreted as relative, within-plate comparisons rather than absolute lipid measurements. Several methodological considerations should be noted when interpreting these findings. First, the use of HepG2 and THP-1 cell lines provides scalability and reproducibility but necessarily limits the representation of the full metabolic capacity of primary hepatocytes and the phenotypic heterogeneity of liver macrophage populations. The elevated AFP expression and reduced CYP3A4 activity relative to primary cells are well-documented features of HepG2-based models [35,36]. Future studies should assess whether the observed glucolipotoxic responses and macrophage remodeling patterns are reproduced in primary hepatocyte or iPSC-derived co-culture systems. Second, the botanical extracts were tested at single concentrations as a proof-of-concept demonstration of model responsiveness. Dose–response characterization and comparison with pharmacological reference compounds (e.g., pioglitazone, resveratrol) will be necessary to establish the quantitative sensitivity and assay specificity of the model for compound evaluation. Third, the 24 h acute challenge is intentionally designed as a short-term stress assay. It captures early metabolic and inflammatory responses but does not replicate the chronic, progressive nature of MASLD or fibrogenic remodeling. Longer or repeated exposure protocols, as well as the incorporation of additional cell types (e.g., hepatic stellate cells for fibrogenic assessment), represent natural extensions of this work. Finally, while immunofluorescence and Live/Dead staining showed no evident necrotic core, optical clearing with confocal imaging would provide more rigorous confirmation of cell viability distribution throughout the spheroid structure.

Despite these limitations, the present model offers a reproducible system that integrates hepatocyte lipid accumulation with macrophage-mediated inflammatory signaling in a defined 3D architecture. By providing two orthogonal, mechanistically informative readouts within a standardized format, the model addresses a gap between simple 2D lipid-loading assays and complex primary-cell microphysiological systems and offers a foundation for investigating hepatic lipid–immune interactions in the context of metabolic stress.

## 5. Conclusions

This study describes the development and characterization of a matrix-free 3D HepG2/THP-1 spheroid co-culture model that enables simultaneous assessment of lipid accumulation and NF-κB-mediated inflammatory activation under glucolipotoxic stress. The model captured dose-dependent responses to palmitic acid, coordinated macrophage transcriptional remodeling consistent with lipid-handling programs, and differential responsiveness to botanical bioactive compounds across the two endpoints. These features support its utility as a reproducible, fit-for-purpose screening tool for early glucolipotoxic responses. Future work should incorporate protein-level readouts, dose–response characterization with pharmacological references, longer exposure protocols, and primary-cell validation to define the broader applicability of this system.

## Figures and Tables

**Table 1:** Antibodies used for immunofluorescence assay.

Reagent	Dilution	Supplier	Product Catalog Number
Mouse anti-human CD14	10 µg/mL	Thermo Fisher Scientific	MA5-14773
Rabbit anti-human albumin	10 µg/mL	Thermo Fisher Scientific	MA5-32531
Goat anti-rabbit secondary antibody, FITC conjugated	4 µg/mL	Thermo Fisher Scientific	A27034
Donkey anti-mouse secondary antibody, TRITC conjugated	4 µg/mL	Thermo Fisher Scientific	A16016

FITC = Fluorescein isothiocyanate; TRITC = tetramethylrhodamine.

**Table 2:** Primer list used for gene expression.

	Gene	Primer Sequence (5′→3′)	Product Length (bp)	Accession N.
Hepatocyte markers	ALB	F: GATGAGATGCCTGCTGACTTGC R: CACGACAGAGTAATCAGGATGCC	147	NM_000477.7
	AFP	F: GCAGAGGAGATGTGCTGGATTG R: CGTGGTCAGTTTGCAGCATTCTG	113	NM_001354717.2
	CYP3A4	F: CCGAGTGGATTTCCTTCAGCTG R: TGCTCGTGGTTTCATAGCCAGC	134	NM_001202855.3
Macrophage markers	CD14	F: CTGGAACAGGTGCCTAAAGGAC R: GTCCAGTGTCAGGTTATCCACC	120	NM_001174104.2
	CD64	F: ATACAGGTGCCAGAGAGGTCTC R: CCAGCTTATCCTTCCACGCATG	149	NM_000566.4
	CD68	F: CGAGCATCATTCTTTCACCAGCT R: ATGAGAGGCAGCAAGATGGACC	136	NM_001251.3
	CD80	F: CTCTTGGTGCTGGCTGGTCTTT R: GCCAGTAGATGCGAGTTTGTGC	136	NM_005191.4
	CD86	F: CCATCAGCTTGTCTGTTTCATTCC R: GCTGTAATCCAAGGAATGTGGTC	154	NM_175862.5
	CD206	F: AGCCAACACCAGCTCCTCAAGA R: CAAAACGCTCGCGCATTGTCCA	121	NM_002438.4
	CD163	F: CCAGAAGGAACTTGTAGCCACAG R: CAGGCACCAAGCGTTTTGAGCT	125	NM_001370145.1
	MARCO	F: GGACAATTTGCGATGACGAGTGG R: CCGACACTGAACATTATCCAGCC	137	NM_006770.4
	CLEC4F	F: CCAAGATACCGAGGCTCGTTCA R: AGGCTTCGGAACAGGTCTTGTC	116	NM_173535.3
	TREM2	F: ATGATGCGGGTCTCTACCAGTG R: GCATCCTCGAAGCTCTCAGACT	151	NM_018965.4
Inflammation and metabolism	Interleukin-8	F: GAGAGTGATTGAGAGTGGACCAC R: CACAACCCTCTGCACCCAGTTT	112	NM_000584.4
	Tumor necrosis factor alpha	F: TCCAACCTTCCCAAACGCCT R: TGCAGGCCACACATTCCTGA	179	NM_000594.4
	Fatty Acid Synthase	F: TTCTACGGCTCCACGCTCTTCC R: GAAGAGTCTTCGTCAGCCAGGA	131	NM_004104
	PRKAA1	F: AGGAAGAATCCTGTGACAAGCAC R: CCGATCTCTGTGGAGTAGCAGT	145	NM_206907
Ref.	TBP	F: TGTATCCACAGTGAATCTTGGTTG R: GGTTCGTGGCTCTCTTATCCTC	124	NM_003194.5
	RPL13	F: CTCAAGGTGTTTGACGGCATCC R: TACTTCCAGCCAACCTCGTGAG	143	NM_012423.4

All primers were designed with PrimerBLAST. Abbreviations: Ref. = reference genes; ALB = albumin; AFP = alpha-fetoprotein; CD = cluster of differentiation; CLEC4F = C-type lectin domain family 4 member F; CYP3A4 = cytochrome P450 family 3 subfamily A member 4; MARCO = macrophage receptor with collagenous structure; PRKAA1 = protein kinase AMP-activated catalytic subunit alpha−1; RPL13 = ribosomal protein L13; TBP = TATA-box binding protein; TREM2 = triggering receptor expressed on myeloid cells 2.

## Data Availability

The original contributions presented in this study are included in the article/Supplementary Material. Further inquiries can be directed to the corresponding author.

## Supplementary Materials

The following supporting information can be downloaded at https://www.mdpi.com/article/10.3390/biomedicines14040792/s1. Figure S1: Morphological development of HepG2/THP-1 spheroids.

## Abbreviations

The following abbreviations are used in this manuscript:

ALB	Albumin
AFP	Alpha-fetoprotein
BSA	Bovine serum albumin
CD	Cluster of differentiation
CLEC4F	C-type lectin domain family 4 member F
CYP3A4	Cytochrome P450 family 3 subfamily A member 4
DAPI	4′,6-diamidino-2-phenylindole
DMEM	Dulbecco’s Modified Eagle’s Medium
DMSO	Dimethyl sulfoxide
DPBS	Dulbecco’s phosphate-buffered saline
EthD-1	Ethidium homodimer-1
FASN	Fatty acid synthase
FBS	Fetal bovine serum
FITC	Fluorescein isothiocyanate
GSE	Grape seed extract
GTE	Green tea extract
HEPES	4-(2-hydroxyethyl)-1-piperazineethanesulfonic acid
HG	High glucose
IL8	Interleukin-8
iPSC	Induced pluripotent stem cell
LAM	Lipid-associated macrophage
LG	Low glucose
MARCO	Macrophage receptor with collagenous structure
MASLD	Metabolic dysfunction-associated steatotic liver disease
MASH	Metabolic dysfunction-associated steatohepatitis
MT	Milk thistle
NAFLD	Non-alcoholic fatty liver disease
NF-κB	Nuclear factor kappa-light-chain-enhancer of activated B cells
PA	Palmitic acid
PMA	Phorbol 12-myristate 13-acetate
PRKAA1	Protein kinase AMP-activated catalytic subunit alpha-1
qPCR	Quantitative polymerase chain reaction
RFU	Relative fluorescence units
RLU	Relative light units
RNA	Ribonucleic acid
RPMI	Roswell Park Memorial Institute medium (RPMI 1640)
RPL13	Ribosomal protein L13
SD	Standard deviation
STR	Short tandem repeat
TBP	TATA-box binding protein
TNFα	Tumor necrosis factor alpha
TREM2	Triggering receptor expressed on myeloid cells 2
TRITC	Tetramethylrhodamine isothiocyanate
