# Supplementary figures and images for: A Matrix-Free 3D Hepatocyte–Macrophage Co-Culture Spheroid Model for Dual Assessment of Lipid Accumulation and NF-κB-Mediated Inflammatory Activation Under Glucolipotoxic Stress
Source: Biomedicines. 2026 Mar 31;14(4):792. doi: 10.3390/biomedicines14040792 (PMC13114099; doi:10.3390/biomedicines14040792)

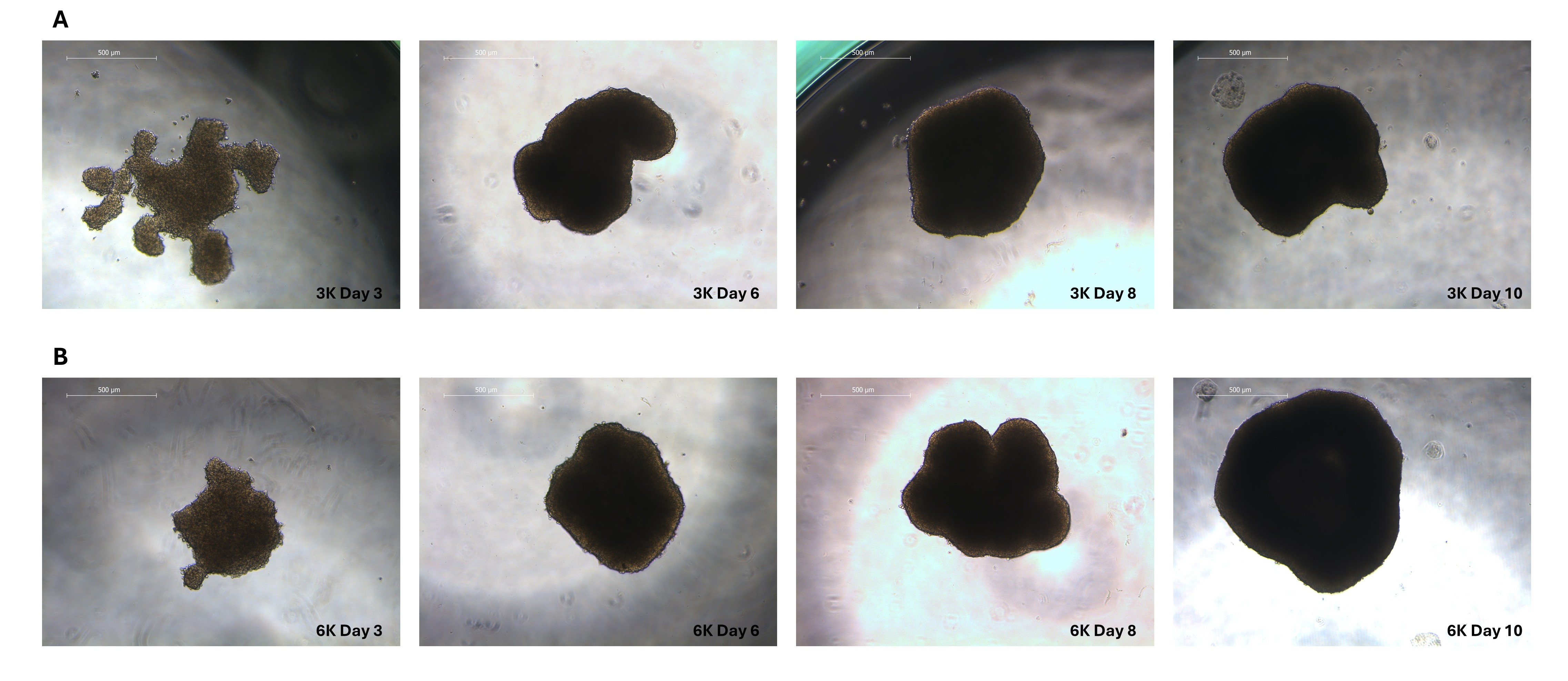

Supplement: Supplementary file 1 [file biomedicines-14-00792-s001.zip › S1 Fig.jpg]
